# Thermodynamics of continuous non-Markovian feedback control

**DOI:** 10.1038/s41467-020-15148-5

**Published:** 2020-03-13

**Authors:** Maxime Debiossac, David Grass, Jose Joaquin Alonso, Eric Lutz, Nikolai Kiesel

**Affiliations:** 1 Faculty of Physics, VCQ, University of Vienna, Boltzmanngasse 5, A-1090 Vienna, Austria; 2 Department of Physics, Friedrich-Alexander-Universität Erlangen-Nürnberg, D-91058 Erlangen, Germany; 3 Institute for Theoretical Physics I, University of Stuttgart, D-70550 Stuttgart, Germany; 4 Present Address: Department of Chemistry, Duke University, Durham, North Carolina 27708 United States

**Keywords:** Optical manipulation and tweezers, Thermodynamics

## Abstract

Feedback control mechanisms are ubiquitous in science and technology, and play an essential role in regulating physical, biological and engineering systems. The standard second law of thermodynamics does not hold in the presence of measurement and feedback. Most studies so far have extended the second law for discrete, Markovian feedback protocols; however, non-Markovian feedback is omnipresent in processes where the control signal is applied with a non-negligible delay. Here, we experimentally investigate the thermodynamics of continuous, time-delayed feedback control using the motion of an optically levitated, underdamped microparticle. We test the validity of a generalized second law which bounds the energy extracted from the system, and study the breakdown of feedback cooling for very large time delays.

## Abstract (web summary)

The second law of thermodynamics cannot be straightforwardly applied to systems with non-Markovian feedback control, and previous extensions to cover that regime have not been tested experimentally. Here, the authors bridge this gap using an optically levitated microsphere in a feedback-cooling system.

## Introduction

The second law of thermodynamics is of fundamental and practical importance^1^. On the one hand, it allows one to predict which transformations are possible in nature. On the other hand, it offers a method for determining the efficiency of a given process by comparing it to its ideal, reversible limit. According to the standard formulation of the second law, no work can be cyclically extracted from a system coupled to a single reservoir at temperature *T*_0_, that is, the power output has to be negative, ${\dot{{\mathcal{W}}}}_{\text{ext}}\ \le \ 0$^1^. However, in the presence of measurement and feedback, this statement of the second law breaks down and positive work can be produced, as exemplified by Maxwell’s and Szilard’s thought experiments^2,3^. For Markovian feedback protocols a refined version of the second law reads ${\dot{{\mathcal{W}}}}_{\text{ext}}\ \le \ {k}_{\text{B}}{T}_{0}{\dot{{\mathcal{I}}}}_{\,\text{flow}}^{\text{mar}\,}$, where *T*_0_ is the bath temperature and ${\dot{{\mathcal{I}}}}_{\,\text{flow}}^{\text{mar}\,}$ is the information flow to the detector, defined as the time variation of the mutual information between a variable and its measured value^4–6^. This inequality has been experimentally verified with colloidal particles^7,8^ and single electrons^9,10^. When the information rate ${\dot{{\mathcal{I}}}}_{\,\text{flow}}^{\text{mar}\,}$ is positive, more work can be extracted from the system than permitted by the usual second law of thermodynamics.

The fact that a control signal cannot be applied instantaneously implies that feedback circuits inevitably exhibit memory effects and are thus non-Markovian. The Markovian approximation is only valid when the delay, i.e., the time between measurement and feedback, is much smaller than the typical timescales of the system. Delayed feedback is widespread in many areas, from chaotic systems to biology^11–15^, emphasizing the crucial need to expand the second law to account for finite memory. However, such generalization is nontrivial. Because of the non-Markovian nature of the feedback, the conventional approach of stochastic thermodynamics^16^ cannot be applied and the usual condition of local detailed balance does not hold. As a result, new contributions to the nonequilibrium entropy production occur, leading to the extended second law for continuous, non-Markovian feedback, ${\dot{{\mathcal{W}}}}_{\text{ext}}\ \le \ {k}_{\text{B}}{T}_{0}{\dot{{\mathcal{S}}}}_{\text{pump}}$, where ${\dot{{\mathcal{S}}}}_{\text{pump}}$ is the entropy pumping rate, which incorporates the effect of the time delay^17–20^. Since ${\dot{{\mathcal{S}}}}_{\text{pump}}\ \le \ {\dot{{\mathcal{I}}}}_{\,\text{flow}}^{\text{mar}\,}$, this is the tightest second-law inequality to date^18^. Despite the omnipresence of delay in feedback processes, a dedicated experimental investigation of this non-Markovian generalization of the second-law inequality is still lacking.

Here, we report the experimental study of the thermodynamics of continuous, non-Markovian feedback control applied to the underdamped center-of-mass (CM) motion of a levitated microsphere^21^. Levitated particles are an ideal experimental platform to explore thermodynamics in small systems^22–27^. We confirm the validity of the generalized second law for time delays spanning two decades and observe the breakdown of the high-quality-factor (high Q) approximation^18^. We establish that the efficiency of the feedback is enhanced when non-Markovian effects are included. We further explore the relation between Markovian and non-Markovian feedback by analyzing how the delay affects the correlations between measurement outcome and velocity of the particle. We finally explore the limitations of feedback cooling and the saturation of the effective CM temperature of the system above the bath temperature for very large delay.

## Results

### Generalized second law

**Fig. 1:**
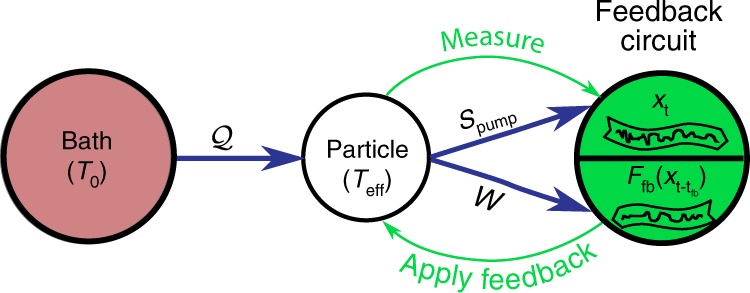
Non-Markovian delayed feedback control. The system consists of a particle (white) in contact with a single heat bath (red) and coupled to a feedback reservoir (green). The feedback loop continuously detects the position *x*_*t*_ of the particle and applies a delayed feedback force ${F}_{\text{fb}}({x}_{t-{t}_{\text{fb}}})$ proportional to the position at time *t* − *t*_fb_. Depending on the time delay *t*_fb_, the feedback cools or heats the system to a steady-state effective temperature *T*_eff_. The blue arrows in the figure correspond to cooling, when the feedback control pumps the entropy ${{\mathcal{S}}}_{\text{pump}}$ out of the system. Energy flows as heat ${\mathcal{Q}}$ from the bath to the particle and is extracted as work ${\mathcal{W}}$ by the feedback circuit (blue arrows). In the case of heating, the energy and entropy flows change direction. In both regimes the generalized second law holds in the form, ${\dot{{\mathcal{W}}}}_{\text{ext}}=-\dot{{\mathcal{W}}}\ \le \ {k}_{\text{B}}{T}_{0}{\dot{{\mathcal{S}}}}_{\text{pump}}$.

We consider a harmonic oscillator in contact with a heat bath at temperature *T*_0_, and subjected to a delayed feedback control that acts as an information reservoir (Fig. 1). Delayed feedback control means here that we acquire information about the oscillator position *x*_*t*_ at time *t* and apply a force ${F}_{\text{fb}}(t)\propto {x}_{t-{t}_{\text{fb}}}$ to manipulate its motion based on the position measured at time *t* − *t*_fb_. The stochastic dynamics of the oscillator is governed by the underdamped Langevin equation^28^,

1 $${\ddot{x}}_{t}+{\Gamma }_{0}{\dot{x}}_{t}+{\Omega }_{0}^{2}{x}_{t}-g{\Gamma }_{0}{\Omega }_{0}{x}_{t-{t}_{\text{fb}}}=\sqrt{\frac{2{\Gamma }_{0}{k}_{\mathrm{{B}}}{T}_{0}}{m}}{\xi }_{t},$$

with *m* the particle mass, *Ω*_0_ its natural frequency, and Γ_0_ the damping coefficient. The quantity *ξ*_*t*_ is a centered Gaussian white noise with $\langle \xi (t)\xi (t^{\prime} )\rangle =\delta (t-t^{\prime} )$. The linear feedback is applied via the force ${F}_{\text{fb}}=-gm{\Gamma }_{0}{\Omega }_{0}{x}_{t-{t}_{\text{fb}}}$ with feedback gain *g* > 0^18^. The mechanical quality factor of the resonator is given by *Q*_0_ = *Ω*_0_∕*Γ*_0_ and the feedback damping rate by *Γ*_fb_ = *gΓ*_0_. It is convenient to introduce the normalized delay *τ* = *t*_fb_*Ω*_0_ and the position of the oscillator normalized to its standard deviation in equilibrium^17–20^. The dynamics of the particle is then fully characterized by a set of dimensionless parameters (*g*, *Q*_0_, *τ*) (Methods). Both the harmonic and Brownian terms in Eq. (1) are Markovian. Memory effects enter only via the delayed feedback force *F*_fb_.

**Fig. 2:**
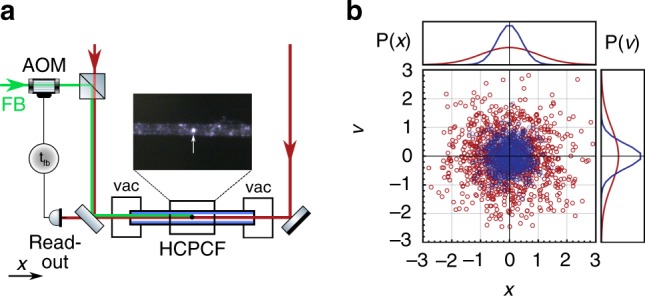
Experimental setup. **a** Two counterpropagating laser beams (red) are coupled into a hollow-core photonic crystal fiber (HCPCF). They form a standing wave that allows for trapping a silica microparticle at an antinode (inset, white arrow). The fiber ends are placed inside vacuum chambers (vac) to control the pressure inside the HCPCF. The particle position along the fiber axis is detected with an interferometric readout. The feedback loop is implemented by delaying the detected signal by a time *t*_fb_. To apply the feedback force, a feedback laser (green) is modulated with the delayed signal via an acousto-optic modulator (AOM). **b** Phase-space distribution *P*(*x*, *v*) of the microparticle derived from the position measurements at *T*_eff_ = *T*_0_ (red circles) and *T*_eff_ < *T*_0_ (blue circles). The respective marginals for the distributions of position and velocity are shown on the top and right panels.

When the feedback force acts to cool the harmonic oscillator, the extracted work ${{\mathcal{W}}}_{\text{ext}}=-{\mathcal{W}}$ is taken to be positive. According to the first law, the energy balance reads $\Delta U={\mathcal{W}}-{\mathcal{Q}}$. As a result, the heat dissipated into the heat bath in the steady state is ${\mathcal{Q}}=-{{\mathcal{W}}}_{\text{ext}}$. On the other hand, the steady-state entropy balance is^17–20^

2 $$-\frac{\dot{{\mathcal{Q}}}}{{k}_{\mathrm{B}}{T}_{0}}=\frac{{\dot{{\mathcal{W}}}}_{\text{ext}}}{{k}_{\mathrm{B}}{T}_{0}}\,{\le}\, {\dot{{\mathcal{S}}}}_{\text{pump}},$$

where ${\dot{{\mathcal{S}}}}_{\text{pump}}$ is the entropy pumping rate, an additional contribution to the entropy production that stems from the feedback control and depends on the delay for non-Markovian protocols. The entropy pumping rate is computed by coarse-graining the harmonic and feedback forces over the position variable^17–20^ (Methods). For a harmonic potential and a linear feedback force, the velocity distribution is a Gaussian (Fig. 2b), $P(v)=\exp \left(-{v}^{2}/2{\sigma }_{v}^{2}\right)/\sqrt{2\pi {\sigma }_{v}^{2}}$ with variance ${\sigma }_{v}^{2}$. The non-Markovian feedback control leads to a cooling of the microparticle, corresponding to negative entropy pumping and extracted work rates, when ${\sigma }_{v}^{2}\,{<}\,1$. By contrast, heating occurs for ${\sigma }_{v}^{2}\,{> }\, 1$.

The validity of inequality (2) can be assessed by comparing the entropy pumping to Markovian bounds. The usual Markovian velocity feedback (VFB) cooling^29,30^, with a feedback force proportional to the instantaneous velocity of the particle, *F*_fb_ ∝ − *v*, is recovered in the limit of *Q*_0_ ≫ *τ* and for *τ* = *π*∕2 +  2*πn*, with *n* an integer. The entropy pumping rate corresponds in this case to ${\dot{{\mathcal{S}}}}_{\text{vfb}}=g/{Q}_{0}$^31^, which we identify as the Markovian information flow ${\dot{{\mathcal{I}}}}_{\,\text{flow}}^{\text{mar}\,}$ (Methods). The high-quality-factor approximation (*Q*_0_ ≫ 1) allows one to map the non-Markovian dynamics to an effectively Markovian Langevin equation, because the motion of the oscillator is essentially coherent on that timescale^18^. The effect of the feedback is then incorporated in a modified damping $\Gamma ^{\prime} ={\Gamma }_{0}(1+g\sin \tau )$ and mechanical frequency $\Omega {^{\prime} }^{2}={\Omega }_{0}^{2}[1-({\Gamma }_{\text{fb}}/{\Omega }_{0})\cos \tau ]$ of the resonator (Supplementary Note 2). The Markovian second law remains valid with these modified parameters. In the high Q approximation, the entropy pumping is given by ${\dot{{\mathcal{S}}}}_{\text{highQ}}=(g/{Q}_{0})\sin \tau$^18^. The approximation is expected to breakdown for larger *τ*, when the Brownian force noise leads to dephasing between the oscillator motion and the feedback signal. This regime can only be correctly described using the generalized second law (2).

### Experimental setup and results

In our experiment, we use an optically levitated microparticle to implement the dynamics of Eq. (1), which holds for any harmonic system with linear feedback control. A standing wave is formed by two counterpropagating laser beams (*λ* = 1064 nm) inside a hollow-core photonic crystal fiber (HCPCF) (Fig. 2a and Methods)^21^. A silica microsphere (969-nm diameter) is trapped at an intensity maximum of the standing wave. The amplitude of the particle motion is sufficiently small to allow for a harmonic approximation of the potential with frequency *Ω*_0_∕2*π* = 404 kHz (Supplementary Note 7). The damping coefficient *Γ*_0_ as well as the bath temperature *T*_0_ = 293 K are determined by the surrounding gas. In our setup, the linear dependence of the damping coefficient Γ_0_ on the environmental pressure allows simple and systematic tuning of this parameter along with the mechanical quality factor *Q*_0_ = *Ω*_0_ ∕ *Γ*_0_. The particle motion along the *x*-axis is detected by interferometric readout of the light scattered by the particle^21^. The signal is fed into a delay line that is digitally implemented, and the output signal serves to control the power of a feedback laser. This laser exerts a radiation pressure force in one direction, accelerating the microparticle proportional to the delayed particle position. The overall amplification of the signal sets the proportionality constant, which is given by *gΩ*_0_*Γ*_0_, where the gain in our experiment is *g* = 0.36 (Supplementary Note 5). The whole feedback circuit has a minimal delay of *t*_fb_ = 2.6 μs, i.e., *τ* = 2.04*π*.

**Fig. 3:**
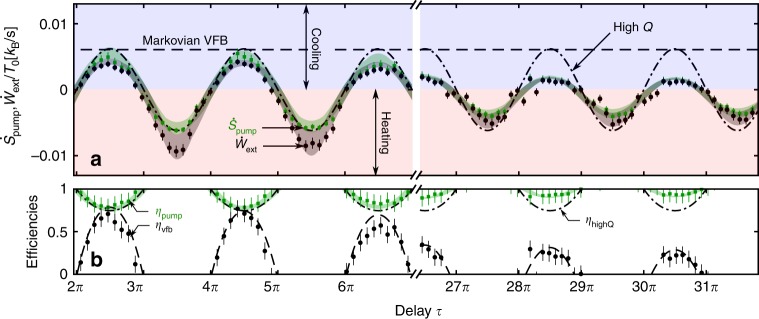
Test of the generalized second law. **a** Experimental entropy pumping (green squares) and extracted work (black circles) rates are plotted as a function of time delay for *Q*_0_ = 55 and *g* = 0.36. The shaded area corresponds to the analytical prediction including experimental parameter drifts (Supplementary Note 8). The error bars represent statistical uncertainties. Theoretical predictions for Markovian entropy bounds to the extracted work are shown for velocity feedback (${\dot{{\mathcal{S}}}}_{\text{vfb}}$, horizontal dashed line) and for the high-quality-factor approximation (${\dot{{\mathcal{S}}}}_{\text{highQ}}$, dashed-dotted line). The blue (red) region corresponds to cooling (heating) of the particle motion. For all delays, the entropy pumping is larger than the extracted work. At the same time, it represents a tighter bound to the extracted work than the Markovian predictions. **b** Feedback efficiencies plotted in the cooling regions only. Experimental pumping efficiency ${\eta }_{\text{pump}}={\dot{{\mathcal{W}}}}_{\text{ext}}/({k}_{\mathrm{B}}{T}_{0}{\dot{{\mathcal{S}}}}_{\text{pump}})$ (green squares) of the generalized second law is compared with the experimental Markovian velocity feedback efficiency ${\eta }_{\text{vfb}}={\dot{{\mathcal{W}}}}_{\text{ext}}/({k}_{\mathrm{B}}{T}_{0}{\dot{{\mathcal{S}}}}_{\text{vfb}})$ (black circles), where ${\dot{{\mathcal{S}}}}_{\text{vfb}}=g/{Q}_{0}$. Theoretical Markovian predictions for *η*_vfb_ (dashed) and ${\eta }_{\text{highQ}}={\dot{{\mathcal{W}}}}_{\text{ext}}/({k}_{\mathrm{B}}{T}_{0}{\dot{{\mathcal{S}}}}_{\text{highQ}})$ (dashed-dotted) with ${\dot{{\mathcal{S}}}}_{\text{highQ}}=(g/{Q}_{0})\sin \tau$ are shown for comparison. Neglecting the non-Markovian behavior leads to a strongly underestimated efficiency of the feedback process.

We first test the extended second law (2) by varying the delay over two decades. Figure 3a demonstrates the validity of the non-Markovian inequality (2) over all relevant timescales. The non-Markovian entropy pumping rate ${\dot{{\mathcal{S}}}}_{\text{pump}}$ (green) is a much more precise upper bound to the extracted work rate (black) than the Markovian pumping rate ${\dot{{\mathcal{S}}}}_{\text{vfb}}$ (horizontal dashed line). In particular, the Markovian result fails to capture the oscillations of the extracted work rate, as well as the heating phases induced by the delay. The high Q approximation (dashed-dotted line) correctly describes the oscillatory behavior of ${\dot{{\mathcal{S}}}}_{\text{pump}}$ for short delays. Yet, it does not account for the oscillation decrease induced by the Brownian force noise for long delays. We already observe significant deviations for a delay of only three oscillation periods with a mechanical quality factor of *Q*_0_ = 55. We have also verified the second law (2) by varying the dissipation via *Q*_0_ (Supplementary Note 9).

The generalized second law (2) is crucial to properly estimate the performance of the feedback cooling. In analogy to heat engines and refrigerators, one may define the efficiency of work extraction ${\eta }_{\text{pump}}={\dot{{\mathcal{W}}}}_{\text{ext}}/({k}_{B}{T}_{0}{\dot{{\mathcal{S}}}}_{\text{pump}})$, which characterizes the conversion of information into extracted work^32^. As shown in Fig. 3b, the corresponding Markovian efficiencies for velocity feedback (*η*_vfb_) and for the high Q approximation (*η*_highQ_) vastly underestimate the feedback efficiency. We note that the pumping efficiency *η*_pump_ and cooling power ${\dot{{\mathcal{W}}}}_{\text{ext}}$ exhibit a trade-off similar to that of heat engines: one is maximal when the other is minimal, and vice versa. By contrast, the velocity feedback efficiency *η*_vfb_ exhibits an opposite dependence on *τ*.

**Fig. 4:**
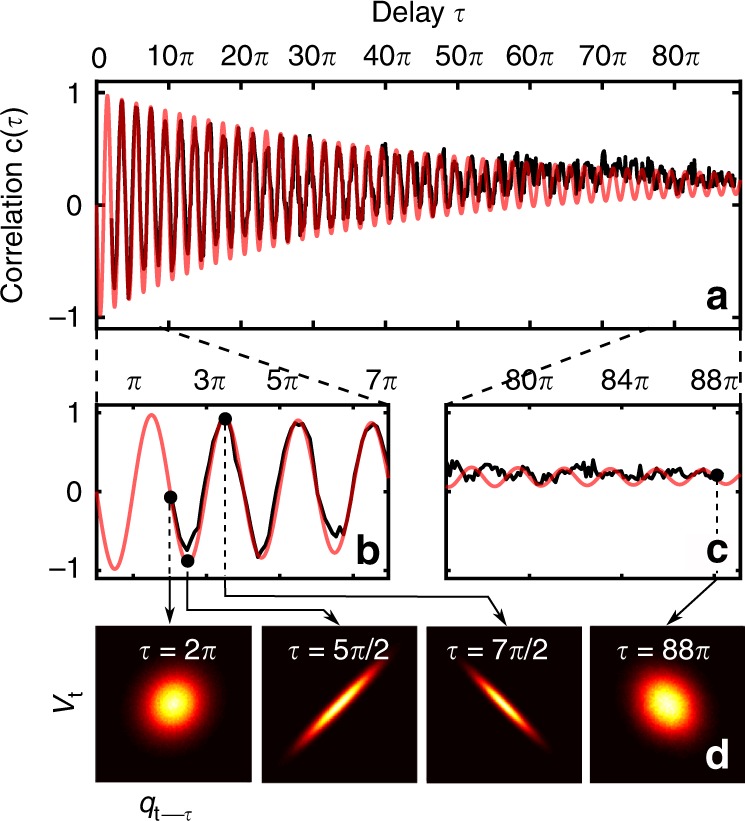
Correlation and colored noise for long delays. **a** Experimental (black) and theoretical (red) correlation functions of the microparticle CM motion as a function of *τ* for *g* = 0.36. Zoom in for short (**b**) and long (**c**) delays. (**d**) Experimental probability distribution *P*(*q*_*t*–*τ*_, *v*_*t*_) for different values of time delay in **b**, **c** indicated by black arrows. The system is still correlated for very long delays (*τ*  = 88*π*) due to resonant driving by colored force noise created by the feedback loop.

We may gain physical insight on the breakdown of the standard second law as shown in Fig. 3 by analyzing the correlations between particle velocity and feedback force, as well as the effective temperature of the system. Cooling is efficient when the feedback force counteracts the motion of the oscillator, in other words, when the velocity *v*_*t*_ of the oscillator and the feedback force (*F* ∝ *x*_*t*−*τ*_) are anticorrelated. Heating occurs when they are correlated. Figure 4 shows the correlation function between the two quantities, *c*(*τ*) =  1∕(*σ*_*q*_*σ*_*v*_)∫∫*y*_*t*_*v*_*t*_*P*(*y*_*t*_, *v*_*t*_)d*y*_*t*_d*v*_*t*_ with *y*_*t*_ = *q*_*t*−*τ*_ (Supplementary Note 4). The delay *τ* has two effects. First, it changes the phase between the mechanical system and the feedback signal deterministically, resulting in the oscillatory behavior of the correlations, and thus the difference between heating and cooling. Second, it allows for stochastic dephasing of the mechanical motion with respect to the feedback signal, which translates into a reduction of the correlations for increasing delay. These correlations do not vanish, however, but asymptotically approach a finite value. For long delays, the oscillator thermalizes due to the damping. The action of the feedback circuit can then be seen as an independent force noise with the spectrum of a white-noise driven harmonic oscillator. The positive correlations occur as a result of the resonant driving of the mechanical motion by the feedback signal.

**Fig. 5:**
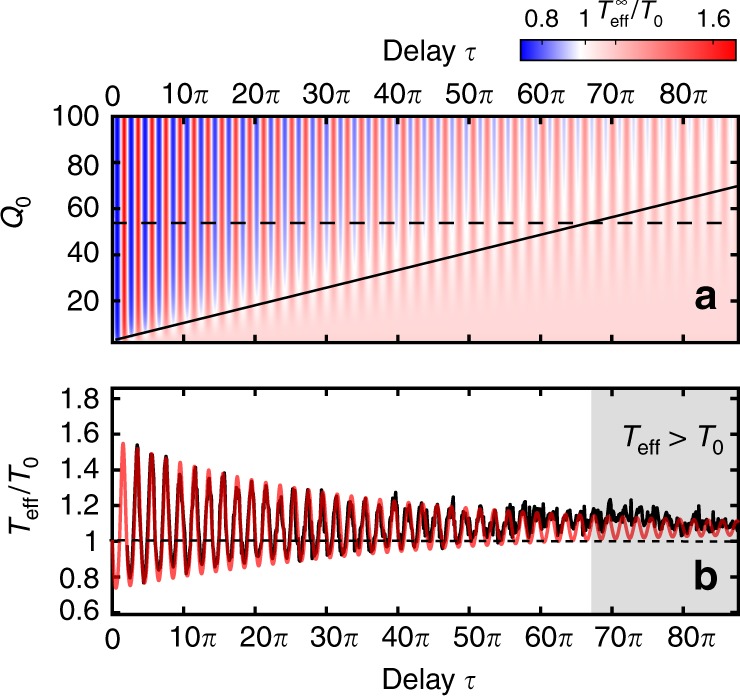
Steady-state effective temperature. **a** Calculated 2D color map of the ratio *T*_eff_∕*T*_0_ of the microparticle motion as a function of *τ* and *Q*_0_ for *g* = 0.36. The black solid line represents the border beyond which cooling is no longer possible. Note that this border is gain dependent. **b** Experimental (black) and calculated (red) normalized effective temperature for *Q*_0_ = 55, corresponding to the dashed horizontal line shown in **a**. The gray-shaded area shows the region with *T*_eff_ > *T*_0_ for very long delays.

Figure 5 displays the ratio of the effective steady-state temperature, ${T}_{\text{eff}}={T}_{0}{\sigma }_{q}^{2}$^18^, and the bath temperature *T*_0_. Figure 5a clearly shows how the action of the feedback is reduced when the mechanical quality factor *Q*_0_ is decreased or the time delay *τ* increased. For all values of *Q*_0_, there is a certain delay *τ*, beyond which cooling is no longer possible (black line). For even longer delays, the effective temperature reaches a constant value, ${T}_{\,\text{eff}\,}^{\infty }/{T}_{0}\approx 1+{g}^{2}/2$, that is independent of *Q*_0_ for weak coupling *g* ≪ *Q*_0_ (Supplementary Note 3). This is in line with the second law that predicts an asymptotic negative work extraction, ${{\mathcal{W}}}_{\,\text{ext}\,}^{\infty }\approx -{g}^{2}/(2{Q}_{0})$ for very long delays. Figure 5b provides a cut for constant *Q*_0_ = 55 through Fig. 5a. The gray-shaded area on the right shows the region where *T*_eff_ > *T*_0_ for our gain *g* = 0.36. Note that this region can be reduced by decreasing the feedback gain. Excellent agreement between theory (red) and data (black) is observed.

## Discussion

In summary, we have performed an extensive experimental study of the second law of thermodynamics in the presence of continuous non-Markovian feedback. Our results constitute an important step toward bridging theoretical developments in stochastic information thermodynamics and more technical applications like continuous feedback. Possible generalizations include the consideration of measurement noise^31^ and the extension to nonlinear potentials and nonlinear feedback (e.g., parametric cooling of levitated nanoparticles^33^). Nonlinear delayed feedback control has thus already proven to be a more robust and effective method for synchronization compared with its linear counterpart^34^. Another avenue of future research is the use of optimal control via Kalman–Bucy filters^20^. Intuitively, one might hope that elaborate filtering methods, like Kalman filters, may overcome the impact of delay that we observe in our simple feedback scenario by an optimal prediction of the instantaneous velocity. However, while this will help to reduce the effect of measurement noise, the effect of stochastic Brownian force noise that occurs during the delay is fundamentally unpredictable. We therefore anticipate no improvement compared with the long-delay results presented in our work.

## Methods

### Normalized form of the equation of motion

After time contraction *t* → *tΩ*_0_ and position normalization *x* → *q* = *x*∕*x*_th_ with ${x}_{\text{th}}=\sqrt{{k}_{\text{B}}{T}_{0}/m{\Omega }^{2}}$ the thermal root mean square amplitude, the Langevin equation can be rewritten in the dimensionless form,

3 $${\ddot{q}}_{t}+\frac{1}{{Q}_{0}}{\dot{q}}_{t}+{q}_{t}-\frac{g}{{Q}_{0}}{q}_{t-\tau }=\sqrt{\frac{2}{{Q}_{0}}}{\xi }_{t},$$

with *Q*_0_ = *Ω*_0_∕*Γ*_0_ the quality factor, *g* = *Γ*_fb_∕*Γ*_0_ the feedback gain, *τ* = *t*_fb_*Ω*_0_ the normalized delay and *ξ*_*t*_ representing a centered Gaussian white noise force with $\langle \xi (t)\xi (t^{\prime} )\rangle =\delta (t-t^{\prime} )$.

### Experimental setup

The particle is trapped in an intensity maximum of the standing wave and oscillates in a harmonic trap. For a laser power of 400 mW in each trapping beam, the mechanical frequency is *Ω*_0_∕2*π* = 404 kHz. The particle CM motion is recorded using a balanced photodiode and roughly 10% of the light transmitted through the HCPCF and that scattered by the particle. The feedback control is implemented via radiation pressure of a second laser beam which is orthogonally polarized and frequency shifted with respect to the trapping laser to avoid interference effects. The feedback force *F*_fb_ is realized in the following steps. The readout signal of the CM motion is bandpass filtered (bandwith: 600 kHz, center frequency: *Ω*_0_∕2*π*) to get rid of technical noise in the detector signal. Then, the signal can be amplified and delayed by an arbitrary time *t*_fb_ with a field programmable gate array. This signal is then used as modulation input for the AOM. A more detailed description of the experimental setup and the feedback control can be found in Supplementary Note 6 and in ref. ^21^

### Thermodynamic quantities

The non-Markovian entropy pumping rate ${\dot{{\mathcal{S}}}}_{\text{pump}}$ can be computed by coarse-graining the harmonic (h) and feedback (fb) forces over the position variable: ${\dot{{\mathcal{S}}}}_{\text{pump}}=-\int \text{d}\,v\left[\overline{{F}_{\text{fb}}(v)}+\overline{{F}_{\text{h}}(v)}\right]{\partial }_{v}P(v)$, where *P*(*v*) is the velocity distribution and $\overline{{F}_{\text{i}}(v)}=\int dx\overline{{F}_{\text{i}}(x,v)}P(x| v)$ is the corresponding coarse-grained forces^17–20^. On the other hand, the Markovian information flow ${\dot{{\mathcal{I}}}}_{\,\text{flow}}^{\text{mar}\,}$ in the case of VFB is given by the time variation of the mutual information between the velocity *v* and the measured value of the velocity *y*: ${\dot{{\mathcal{I}}}}_{\,\text{flow}}^{\text{mar}}=\int \text{d}v\text{d}\,y{\partial }_{v}J(v)\,\text{ln}\,[P(v,y)/P(v)P(y)]$ with the velocity probability current *J*(*v*)^18–20^. This quantity diverges for error-free feedback^31^. We thus identify the Markovian entropy bound with the Markovian limit of ${\dot{{\mathcal{S}}}}_{\text{pump}}\to {\dot{{\mathcal{S}}}}_{\text{vfb}}=g/{Q}_{0}$^18–20^. For a harmonic potential and a linear feedback force, the velocity distribution is a Gaussian, $P(v)=\exp \left(-{v}^{2}/2{\sigma }_{v}^{2}\right)/\sqrt{2\pi {\sigma }_{v}^{2}}$, with variance ${\sigma }_{v}^{2}$. The entropy pumping rate is then explicitly ${\dot{{\mathcal{S}}}}_{\text{pump}}=(1-{\sigma }_{v}^{2})/({Q}_{0}{\sigma }_{v}^{2})$ and the work extraction rate reads ${\dot{{\mathcal{W}}}}_{\text{ext}}/({k}_{\mathrm{B}}{T}_{0})=(1-{\sigma }_{v}^{2})/{Q}_{0}$ (Supplementary Note 1). We experimentally obtain the velocity variance needed to verify the generalized second law as follows: for a given time delay *τ*, a position time trace *x*(*t*) is recorded, filtered with a bandwidth of 3*Γ*_0_ via post processing, and normalized with the standard deviation of the feedback beam turned off, to find the normalized position *q*(*t*). The velocity $v=\dot{q}$ is then numerically calculated with the finite difference approximation and the variance ${\sigma }_{v}^{2}$ computed.

## Supplementary information

Supplementary Information

## Data Availability

The datasets generated in the current study are available from MD on reasonable request.
