# Supplementary material for: Thermodynamics of continuous non-Markovian feedback control
Source: Nat Commun. 2020 Mar 13;11:1360. doi: 10.1038/s41467-020-15148-5 (PMC7070085; doi:10.1038/s41467-020-15148-5)
Supplement: Supplementary file 1 — Supplementary Information [file 41467_2020_15148_MOESM1_ESM.pdf]

Supplementary Information - Thermodynamics of continuous non-Markovian feedback control

Debiossac et al.

Supplementary Note 1: Derivation of $\dot{\mathcal{S}}_{\text{pump}}$ and $\dot{W}_{\text{ext}}$

The relevant thermodynamics quantities are here derived from the Fokker-Planck equation associated to the Langevin equation (3),

$$\frac{\partial P(v)}{\partial t} = -\frac{\partial J(v)}{\partial v} \tag{1}$$

with $P(v)$ the velocity probability distribution and

$$J(v) = J_{\text{irr}}(v) - (\overline{q} - \frac{g}{Q_0}\overline{q_{t-\tau}})P(v) \tag{2}$$

the current composed of the irreversible current,

$$J_{\text{irr}}(v) = -\frac{v}{Q_0}P(v) - \frac{1}{Q_0}\frac{\partial P(v)}{\partial v}. \tag{3}$$

We start from the definition of the pumping entropy given in the Methods, $\dot{\mathcal{S}}_{\text{pump}} = -\int \mathrm{d}v[\overline{F_{\text{fb}}(v)} + \overline{F_{\text{h}}(v)}]\partial_v P(v)$, which can be rewritten as

$$\dot{\mathcal{S}}_{\text{pump}} = -\int \mathrm{d}v[\frac{g}{Q_0}\overline{q_{t-\tau}} + \overline{q}]\partial_v P(v) \tag{4}$$

In the steady state, $J(v) = 0$, such that we have from Supplementary Equation (2),

$$\dot{\mathcal{S}}_{\text{pump}} = -\int \mathrm{d}v \frac{J_{\text{irr}}}{P(v)}\partial_v P(v) \tag{5}$$

or equivalently,

$$\dot{\mathcal{S}}_{\text{pump}} = \frac{1}{Q_0}\int_{-\infty}^{\infty} \mathrm{d}v \left(v + \frac{1}{P(v)}\frac{\partial P}{\partial v}\right)\frac{\partial P}{\partial v}. \tag{6}$$

Now assuming a Gaussian distribution $P(v) = \frac{1}{\sqrt{2\pi}\sigma_v}e^{-v^2/2\sigma_v^2}$ and using the analytical expression from Gaussian integrals of the type $\int_{-\infty}^{\infty} x^{2n}e^{-\alpha x^2}dx = \sqrt{\frac{\pi}{\alpha}}\frac{(2n-1)!!}{(2\alpha)^n}$, we arrive at the expression given in the Methods:

$$\dot{\mathcal{S}}_{\text{pump}} = \frac{1-\sigma_v^2}{Q_0\sigma_v^2} \tag{7}$$

Following Ref. [1], the velocity variance can now be obtained explicitly as

$$\sigma_v^2 = \frac{1}{2}\frac{1}{(\omega_2^2 - \omega_1^2)}\left(\frac{y_1\omega_1}{x_1} - \frac{y_2\omega_2}{x_2}\right) \tag{8}$$

with the quantities

$$\begin{aligned} \omega_{1,2} &= \left(1 - \frac{1}{2Q_0^2} \pm \frac{1}{Q_0}\sqrt{g^2 - 1 + \frac{1}{4Q_0^2}}\right)^{1/2} \\ x_i &= \cos\left(\frac{\omega_i\tau}{2}\right) + h(\omega_i)\sin\left(\frac{\omega_i\tau}{2}\right) \\ y_i &= h(\omega_i)\cos\left(\frac{\omega_i\tau}{2}\right) - \sin\left(\frac{\omega_i\tau}{2}\right) \\ h(\omega_i) &= -\frac{1}{1-\omega_i^2 - \frac{g}{Q_0}}\frac{\omega_i}{Q_0}. \end{aligned} \tag{9}$$

Notice that Supplementary Equation (8) differs with the one obtained in Ref. [1] by a factor $Q_0/2$. The same factor will also appear for σ_x^2. This is due to the normalization of the equation of motion. In the approach of Ref. [1], the normalization constant is picked to simplify the stochastic equation while here the constant is chosen to normalize the degrees of freedom of the particle, which also guarantees faster numerical convergence.

Supplementary Equation (7) in combination with Supplementary Equation (8) provides us with the expression of the pumping entropy as a function of (g, Q_0, τ). The definition of the pumping entropy, as well as heat, appears naturally when one writes the entropy balance from the Shannon entropy $\mathcal{S}(t) = -\int dvP(v)\ln P(v)$,

$$\frac{d\mathcal{S}}{dt} = \dot{\mathcal{S}}_i - \frac{\dot{\mathcal{Q}}}{T_0} - \dot{\mathcal{S}}_{\text{pump}} \tag{10}$$

with $\dot{\mathcal{S}}_i = Q_0\int dv\frac{[J_{\text{irr}}]^2}{P(v)}$, $\frac{\dot{\mathcal{Q}}}{T_0} = -\int vJ_{\text{irr}}dv$ and $\dot{\mathcal{S}}_{\text{pump}} = -\int \mathrm{d}v\frac{J_{\text{irr}}}{P(v)}\partial_v P(v)$. Following the same strategy as for the pumping entropy, we obtain the explicit expressions for the extracted work $\frac{\dot{\mathcal{W}}_{\text{ext}}}{T_0} = \frac{1-\sigma_v^2}{Q_0}$ and the positive entropy $\dot{\mathcal{S}}_i = \frac{(1-\sigma_v^2)^2}{Q_0\sigma_v^2}$.

Supplementary Note 2: High Q approximation (high Q)

In the high Q approximation the particle motion is essentially a sinusoidal signal $x_t \propto \sin(\Omega_0 t)$. The delay feedback term can be expanded as

$$x_{t-t_{\mathrm{fb}}} = x\cos\tau - \frac{1}{\Omega_0}\dot{x}\sin\tau \tag{11}$$

The equation of motion (1) then becomes

$$\ddot{x}_t + \Gamma_0\dot{x}_t + \Omega_0^2 x_t - g\Gamma_0\Omega_0(x\cos\tau - \frac{1}{\Omega_0}\dot{x}\sin\tau) = \sqrt{\frac{2\Gamma_0 k_B T_0}{m}}\xi_t \tag{12}$$

or

$$\ddot{x}_t + \Gamma'\dot{x}_t + \Omega'^2 x_t = \sqrt{\frac{2\Gamma_0 k_B T_0}{m}}\xi_t \tag{13}$$

with the modified damping rate $\Gamma' = \Gamma_0(1 + g\sin\tau)$ and mechanical frequency $\Omega'^2 = \Omega_0^2(1 - g\Gamma_0/\Omega_0\cos\tau)$. The dynamics of the microparticle described by Supplementary Equation (13) is Markovian.

In the high Q approximation, the entropy pumping $\dot{\mathcal{S}}_{\mathrm{highQ}}$ is obtained by Taylor expanding Supplementary Equation 7 in the limit $1/Q_0 \to 0$ and keeping only the first term, leading to $\dot{\mathcal{S}}_{\mathrm{highQ}} = (g/Q_0)\sin\tau$. Now, by choosing delays such that $\tau = \pi/2 + 2\pi n$ with n an integer, we arrive at the expression of the velocity feedback entropy pumping, $\dot{\mathcal{S}}_{\mathrm{vfb}} = g/Q_0$, which we identify with the Markovian information flow $\dot{\mathcal{I}}^{\mathrm{mar}}_{\mathrm{flow}}$.

Supplementary Note 3: Derivation of the asymptotic temperature $T^{\infty}_{\mathrm{eff}}$

We define the effective configurational temperature by $T_{\mathrm{eff}}/T_0 = \sigma_q^2$. For very long delays, the position variance is given by $\sigma_q^2 = \frac{1}{2Q_0\sqrt{1-\frac{g^2}{Q_0^2}}}\frac{1}{\mathrm{Im}(\omega_1)}$ with $\mathrm{Im}(\omega_1) = \frac{\sqrt{2}}{2}\sqrt{\frac{1}{2Q_0^2} - 1 + \sqrt{1 - \frac{g^2}{Q_0^2}}}$, see [1]. Those asymptotic expressions are valid when $Q_0 > 1/2$ and $|g/Q_0| < \frac{1}{Q_0}\sqrt{1 - \frac{1}{4Q_0^2}}$, which is the case in our experiment. In the case $g \ll Q_0$, we can Taylor expand σ_q^2 to obtain

$$T_{\mathrm{eff}}/T_0 \approx 1 + \frac{1}{2}(1 + 1/Q_0^2)g^2 + ... \tag{14}$$

which, for $Q_0 \gg 1$, can be well approximated by $T_{\mathrm{eff}}/T_0 \approx 1 + \frac{1}{2}g^2 + ...$ For very long delays, the feedback always heats the motion of the particle.

Supplementary Note 4: Derivation of the correlation function $c(\tau)$ for long delays

The correlation function $c(\tau)$ is defined as $c(\tau) = \frac{1}{\sigma_q\sigma_v}\iint q_{t-\tau}v_t P(q_{t-\tau}, v_t)dq_{t-\tau}dv_t$ where $P(q_{t-\tau}, v_t)$ is the probability distribution between the delayed position and the velocity at time t. Integrating the probability distribution $P(q, q_{t-\tau}, v_t)$, see Eq. (105) in Ref. [1], along q gives

$$P(q_{t-\tau}, v_t) = \int P(q, q_{t-\tau}, v)dq = \frac{g}{\pi Q_0}\frac{1}{\sqrt{g^2\sigma_q\sigma_v - (1-\sigma_v)^2}}\exp\left(\frac{g}{Q_0}\frac{g\sigma_q v^2 + 2(1-\sigma_v)vq_{t-\tau} + g\sigma_v q_{t-\tau}^2}{(1-\sigma_v)^2 - g^2\sigma_q\sigma_v}\right) \tag{15}$$

which allows us to compute the correlation function

$$c(\tau) = \frac{1}{g}\frac{1}{\sigma_q\sigma_v}(\sigma_v^2 - 1) \tag{16}$$

For very long delays, the position and velocity variances are given by $\sigma_q \to \frac{1}{2Q_0\sqrt{1-\frac{g^2}{Q_0^2}}}\frac{1}{\mathrm{Im}(\omega_1)}$ and $\sigma_v \to \frac{1}{2Q_0}\frac{1}{\mathrm{Im}(\omega_1)}$ with $\mathrm{Im}(\omega_1) = \frac{\sqrt{2}}{2}\sqrt{\frac{1}{2Q_0^2} - 1 + \sqrt{1 - \frac{g^2}{Q_0^2}}}$. Those asymptotic expressions are valid when $Q_0 > 1/2$ and $|g/Q_0| < \frac{1}{Q_0}\sqrt{1 - \frac{1}{4Q_0^2}}$, which is the case in our experiment. Finally, we can Taylor expand $c(\tau)$ for $g \ll Q_0$,

$$c(\tau) \approx \frac{1}{2}g + \frac{1}{8}g^3 + ... \tag{17}$$

It is remarkable that for large delays the correlation function depends only on the feedback gain.

Supplementary Note 5: Extraction of feedback gain g and quality factor Q_0 from experimental data

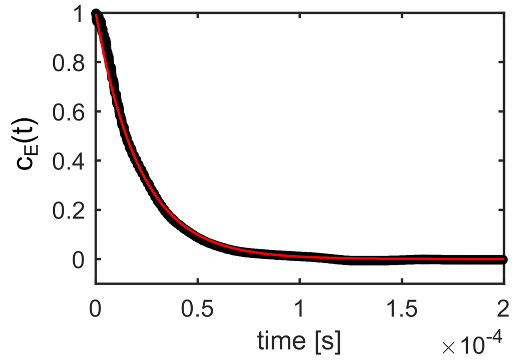

Supplementary Figure 1: Energy autocorrelation function c_E as a function of time. Black circles represent experiment and the red line the fit. We extract from the fit $\Gamma_0/2\pi = 7.37$ kHz.

Here we explain how the feedback gain g and the quality factor Q_0 are extracted from the data. For each delay τ, a time trace $x(t)$ with feedback is recorded with a sampling rate of 10 Ms and a total time of 1s. The time trace is then normalized with the standard deviation without feedback, x_{th}, leading to the normalized position $q(t) = x(t)/x_{\mathrm{th}}$. The normalized time trace is then filtered around the mechanical frequency ν_0 where ν_0 is obtained from the power spectrum density of $x_{\mathrm{th}}(t)$.

The quality factor is defined as $Q_0 = \Omega_0/\Gamma_0$. The damping rate Γ_0 is extracted from the energy autocorrelation function $c_E(t) = \frac{\langle E(t)E(0)\rangle}{\langle E^2\rangle}$ with $E(t) \propto \Omega_0^2 x_{\mathrm{th}}^2 + v_{\mathrm{th}}^2$ and $\Omega_0 = 2\pi\nu_0$. The velocity time trace $v_{\mathrm{th}} = \dot{x}_{\mathrm{th}}$ is obtained by numerical differentiation of the position time trace. For $\Omega_0 > \Gamma_0/2$, the normalized energy autocorrelation of a Brownian particle in an harmonic trap is given by $c_E(t) = e^{-\Gamma_0 t}$ [2]. In Supplementary Figure 1 we show the autocorrelation function corresponding to the time trace x_{th} together with the exponential fit. We have in this case $\Omega_0/2\pi = 404$ kHz and $\Gamma_0/2\pi = 7.37$ kHz, leading to $Q_0 = 55$.

The feedback gain is obtained using two different methods depending if the 2nd law rates are studied as a function of the delay or the quality factor. When the delay is varied, the feedback gain is extracted by fitting T_{eff} using the analytical result $T_{\mathrm{eff}} = \sigma_v^2$ from Supplementary Equation 8. From the fit we get $g = 0.36$. The result of the fit and the experimental data are shown on Fig. 5b of the main text. When the delay is fixed but the quality factor changes, the feedback gain is calculated with $g = \Gamma_{\mathrm{fb}}/\Gamma_0$ where Γ_{fb} is obtained from the energy autocorrelation function of the time trace $q(t)$.

Supplementary Note 6: Detailed description of the feedback loop

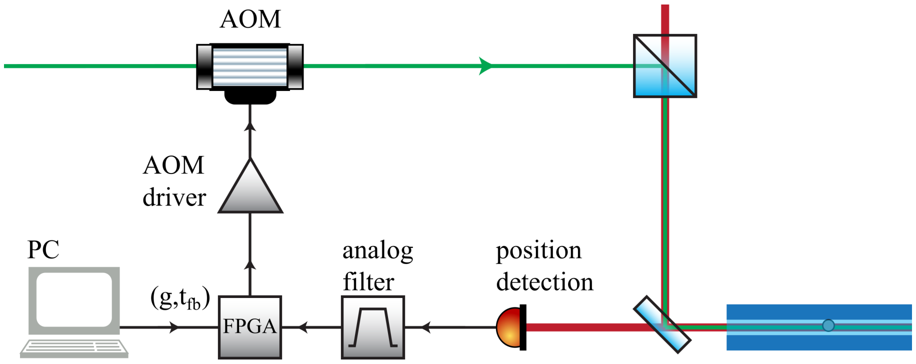

Supplementary Figure 2: The detector voltage (position detection), which is proportional to the particle x displacement, is bandpass filtered with an analog circuit to suppress technical noise (analog filter). The filtered signal is processed by an FPGA which is controlled by a computer (PC). Essentially, we can delay the signal by an amount t_{fb} and multiply it with a gain g. The signal from the FPGA is sent to the modulation input of the AOM driver and hence, causes a modulation of the feedback laser.

Figure 2 illustrates the experimental implementation of the feedback control with emphasis on the circuitry. The position detection generates a voltage which is proportional to the particle displacement along x-direction. A home-made analog bandpass filter is used to suppress technical noise below (mainly of acoustic nature) and above (laser noise) the mechanical frequency. We choose an active, multiple feedback bandpass filter with a center frequency at $f_0 = 400$ kHz, a full width at half maximum bandwidth of $\Delta f = 600$ kHz and we used a AD817 operational amplifier from Analog Devices.

The key element of our feedback circuitry is a field programmable gate array (FPGA) that allows real-time signal processing. Currently, we are using a PXIe-7965 (Virtex 5 based FPGA) from National instruments in combination with a NI-5781 transceiver adapter module, also from National Instruments. The program running on the FPGA has two main functions: (1) Introduction of a discrete delay t_{fb} which can be adjusted in steps of $\Delta t_{\text{fb}} = 100$ ns between input and output signal. (2) Multiplication of the input signal with a gain factor g (note that the gain we set in the FPGA is proportional to, but not the same as the gain defined in the main text). This can be used to control the amplitude of the feedback signal. The values of (t_{fb}, g) are parameters in the FPGA program and can be externally controlled by a computer (PC). In a last step, the output of the FPGA is connected to the modulation input of the AOM driver.

The minimum delay in our setup has be determined in the following way: After the detector we send a square signal composed of 4 oscillations at a frequency of 400 kHz, separated by a time much longer than the delay to be measured. The signal then propagates through the feedback circuit (filter+FPGA) and is sent to the AOM driver controlling the feedback light. The signal from the feedback beam is finally recorded on the detector and we measure a minimum delay of 2.6 μs.

Supplementary Note 7: Normality test of the position and velocity distributions

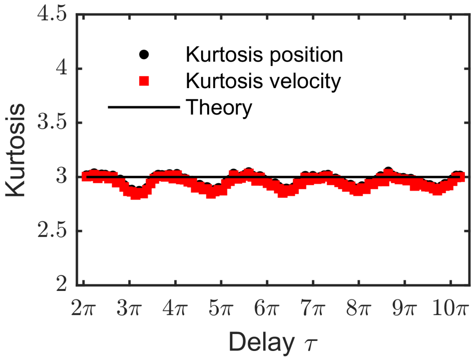

Supplementary Figure 3: Kurtosis as a function of the delay calculated for the position and velocity distributions. Here $Q_0 = 55$ and $g = 0.36$

Here we show that the position and velocity distributions exhibit Gaussian distribution by calculating the kurtosis [4], of each distributions as a function of the delay τ, see Supplementary Figure 3. The kurtosis is evaluated for a time trace $x(t)$ composed of N values using $\text{kur}_x = \frac{1}{N}\sum_{i=1}^{N}(x_i - \bar{x})^4 / \left[\frac{1}{N}\sum_{i=1}^{N}(x_i - \bar{x})^2\right]^2$. Similarly, the kurtosis for the velocity, kur_v, is obtained by replacing the position by the velocity in the previous formula. For a Gaussian distribution, the kurtosis equals 3. It is worth mentioning that the largest deviation from normality comes from delays corresponding to the driving mode. Indeed, in this case the particle is more likely to probe non-linear parts of the optical potential.

Supplementary Note 8: Experimental drifts

We describe in the section the experimental drifts used in Fig. 3 of the main text. The main source of drifts in our setup come from pressure inside the vacuum chamber and power fluctuations due to the 50% coupling efficiency of the laser inside the hollow core fiber. Over the time acquisition of the entire data (30 min), the pressure has increased by 2% and the trapping laser and feedback laser beams fluctuate typically by 5% in transmission through the hollow core fiber. The laser has, however, a power stability of 0.7%. To account for those drifts, we have plotted the shaded area from theoretical predictions using a gain $g \pm 2\sigma_g$ where $\sigma_g/g = \sqrt{(\sigma_{P_{\text{fb}}}/P_{\text{fb}})^2 + (\sigma_{\Gamma_0}/\Gamma_0)^2 + (\sigma_{\Omega_0}/\Omega_0)^2} = \sqrt{(0.05)^2 + (0.02)^2 + (0.025)^2} = 6\%$ and $g \propto \frac{P_{\text{fb}}}{\Gamma_0 \Omega_0}$. We have also included an error in the value of the delay τ due to the fluctuations of Ω_0, $\sigma_\tau/\tau = \sigma_{\Omega_0}/\Omega_0 = 2.5\%$. This is taken into account in the shaded area of Fig. 3 by feeding the theoretical predictions with $\tau \pm 2\sigma_\tau$.

Supplementary Note 9: Test of the generalized second law by varying the quality factor

In Supplementary Figure 4 we have tested the validity of the generalized second law (2) as a function of the quality factor Q_0 of the oscillator for two different delays. For a short delay, $\tau = 5\pi/4$ in panel a), the pumping entropy (green squares) follows the prediction of high Q approximation (dashed-dotted line). This is expected since we are in the regime where the high Q approximation holds, $\tau \ll Q_0$. On the other hand, for a delay $\tau = 5\pi/4 + 2\pi \times 9$, the high Q approximation breaks down and the behavior of the pumping entropy and the extracted work can only been correctly reproduced with the generalized second law (green and dark shaded areas). Notice that in panel a) of Supplementary Figure 4, for $Q_0 = 30$, the theoretical prediction for the pumping entropy (green shaded area) starts to deviate already from the high Q approximation. The discrepancy would be even more pronounced with smaller quality factors.

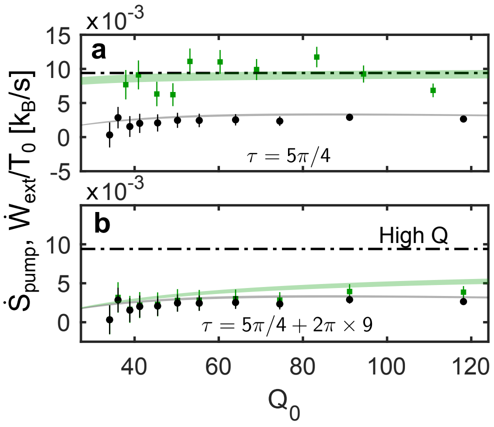

Supplementary Figure 4: **a)** Entropy pumping (green squares) and extracted work (black circles) as a function of the quality factor Q_0 for a feedback gain $g = 0.0094Q_0$ and a delay $\tau = 5\pi/4$. The shaded area corresponds to the analytical prediction including experimental parameter drifts. The error bars represent statistical uncertainties. The horizontal dashed-dotted line refers to the high-quality-factor approximation. **b)** Same as a) but for a much longer delay, $\tau = 5\pi/4 + 2\pi \times 9$.

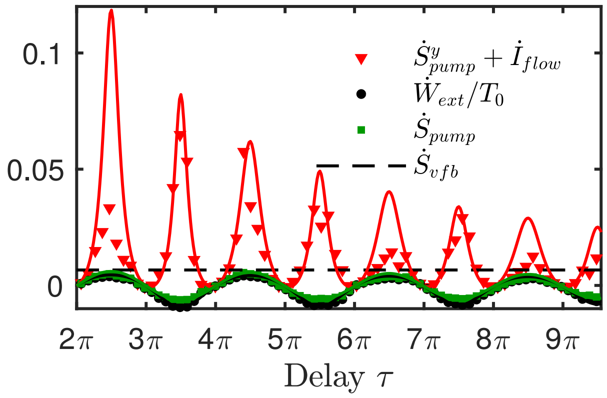

Supplementary Figure 5: Comparison between the entropy pumping and the non-Markovian bound $\dot{\mathcal{S}}^y_{\rm pump}+\dot{\mathcal{I}}_{\rm flow}$ [Supplementary Equation 18] as a function of the delay for $Q_0 = 55$ and $g = 0.36$. Symbols are experimental data and lines theoretical predictions. Data and lines for the entropy pumping and the extracted work are taken from Fig. 3.

Supplementary Note 10: Comparison of the entropy pumping and a non-Markovian bound

In our study we have compared the non-Markovian entropy pumping with Markovian bounds, given by $\dot{\mathcal{S}}_{\rm vfb}$, and $\dot{\mathcal{S}}_{\rm highQ}$. However, it is also interesting to ask if some non-Markovian bounds exist and how they perform against their Markovian counterpart [5–7]. One such answer is given in Ref. [1], Eqs. (68) and (112):

$$\dot{\mathcal{S}}_{\rm pump} \le \dot{\mathcal{S}}^y_{\rm pump} + \dot{\mathcal{I}}_{\rm flow} = \frac{1}{Q_0}(1-\sigma_v^2)\frac{g^2\sigma_x^2 + 1 - \sigma_v^2}{g^2\sigma_x^2\sigma_v^2 - (1-\sigma_v^2)^2} \tag{18}$$

where $\dot{\mathcal{S}}^y_{\rm pump} = Q_0\frac{(\sigma_v^2-1)(\sigma_v^2-\sigma_x^2)}{g^2\sigma_x^2\sigma_v^2-(1-\sigma_v^2)^2}$ is an additional entropy pumping contribution induced by the coarse-graining over x (see Eq. (65) in Ref. [1]) and $\dot{\mathcal{I}}_{\rm flow} = \frac{1}{Q_0}\frac{(1-\sigma_v^2)(Q_0^2(\sigma_v^2-\sigma_x^2)+g^2\sigma_x^2+1-\sigma_v^2)}{g^2\sigma_x^2\sigma_v^2-(1-\sigma_v^2)^2}$ is the non-Markovian information flow rate between $y = x_{t-\tau}$ and v_t, defined as $\dot{\mathcal{I}}_{\rm flow} = \int {\rm d}v{\rm d}y\partial_v J(v)\ln\frac{P(v|y)}{P(v)P(y)}$, see Eqs. (59) and (105) in Ref. [1]. Note that the inequality $\dot{\mathcal{S}}_{\rm pump} \le \dot{\mathcal{I}}_{\rm flow}$ is not satisfied in our system, contrary to Ref. [3] where the harmonic force $F_{\rm harm} = -m\Omega_0^2 x$ is not present, resulting in $\dot{\mathcal{S}}^y_{\rm pump} = 0$.

In Supplementary Figure 5 we have plotted the non-Markovian bound $\dot{\mathcal{S}}^y_{\rm pump}+\dot{\mathcal{I}}_{\rm flow}$, the pumping entropy and the extracted work from Fig. 3 of the main text. Clearly the non-Markovian bound is a much worse bound compared to the pumping entropy. Also, for most of the delays, except around $\tau \approx \pi + n\pi$ with n an integer, even the Markovian bound $\dot{\mathcal{S}}_{\rm vfb}$ is a much tighter bound than the non-Markovian bound.

* Electronic address: maxime.debiossac@univie.ac.at

Supplementary References

[1] M. Rosinberg, T. Munakata, and G. Tarjus, Stochastic thermodynamics of langevin systems under time-delayed feedback control: Second-law-like inequalities, *Phys. Rev. E* **91**, 042114 (2015).
[2] Y. Mishin and J. Hickman, Energy spectrum of a Langevin oscillator, *Phys. Rev. E* **94**, 062151 (2016).
[3] J. M. Horowitz and H. Sandberg, Second-law-like inequalities with information and their interpretations. *New J. Phys.* **16,** 125007 (2014).
[4] K. P. Balanda and H. MacGillivray, Kurtosis: a critical review, *Am. Stat.* **42**, 111119 (1988).
[5] J. M. Horowitz and S. Vaikuntanathan, Nonequilibrium detailed fluctuation theorem for repeated discrete feedback, *Phys. Rev. E* **82**, 061120, (2010).
[6] S. Ito and T. Sagawa, Information thermodynamics on causal networks, *Phys. Rev. Lett.* **111**, 180603, (2013).
[7] R. E. Spinney, J. T. Lizier, and M. Prokopenko, Transfer entropy in physical systems and the arrow of time, *Phys. Rev. E* **94** (2016).
